# Nano-selenium mitigates antibiotic resistance in paddy ecosystems via microbiome remodeling and environmental filtering shifts

**DOI:** 10.1128/aem.02231-25

**Published:** 2026-02-27

**Authors:** Xiaorong Zhang, Qiaobing Luo, Zongqiang Gong, Huimin Yang, Xin Chen, Boshi Wang, Meng Yuan, Yue Chen, Yanjie Jia, Shuhai Guo

**Affiliations:** 1 CAS Key Laboratory of Forest Ecology and Silviculture, Institute of Applied Ecology, Chinese Academy of Sciences 74763 , Shenyang, People's Republic of China; 2 National-Local Joint Engineering Laboratory of Contaminated Soil Remediation by Bio-physicochemical Synergistic Process, Shenyang, People's Republic of China; 3 School of Environmental Science, Liaoning University 12440, Shenyang, People's Republic of China; 4 University of Chinese Academy of Sciences 74519, Beijing, People's Republic of China; 5 Key Laboratory of Conservation Tillage and Ecological Agriculture, Liaoning, People's Republic of China; 6 School of Municipal and Environmental Engineering, Shenyang Jianzhu University 47826, Shenyang, People's Republic of China; University of Georgia Center for Food Safety, Griffin, Georgia, USA

**Keywords:** antibiotic resistance genes, AG-SeNPs, functional prediction, microbial assembly

## Abstract

The dissemination of antibiotic resistance genes (ARGs) in paddy ecosystems poses a serious threat to environmental health. A pot experiment was conducted to assess the efficacy of alkyl glycoside-stabilized selenium nanoparticles (AG-SeNPs) in mitigating ARG abundance within the soil, phyllosphere, and rice grains. Functional prediction, null model analysis, variance partitioning, and structural equation modeling were employed to identify ARG hosts, key metabolic pathways, and environmental drivers of ARG dynamics. Results showed that foliar application of AG-SeNPs (30 g ha^−1^) reduced ARGs by 5.13 × 10^6^ copies g^−1^, 2.28 × 10^7^ copies g^−1^, and 1.25 × 10^6^ copies g^−1^ in the rhizosphere soil, phyllosphere, and grains, respectively. *TetPA* and *tetGF* were dominant ARGs, predominantly associated with *Mariniphaga anaerophila*, *Sediminibacter magnilacihabitans*, and *Limnospira fusiformis*. ARG attenuation was linked to enhanced ABC transporter activity and suppressed purine metabolism and ribosome function, thereby reducing intracellular antibiotic pressure and limiting ARG expression in soil microbes. In the phyllosphere, activation of two-component systems modulated stress responses and antimicrobial resistance pathways, constraining horizontal gene transfer. Nano-selenium increased heterogeneous selection in the phyllosphere, enhancing deterministic filtering of ARG hosts and restructuring microbial communities. Environmental factors explained 42.81% of ARG variation, wherein selenium accumulation in leaves directly reduced ARG abundance, and soil pH, electrical conductivity, and organic matter indirectly influenced ARG dynamics through microbial community restructuring. These findings highlight that AG-SeNPs mitigate ARGs through an environmentally mediated, microbially driven cascade, offering a promising strategy for antibiotic resistance control in agricultural systems.

**IMPORTANCE:**

The dissemination of antibiotic resistance genes within agricultural soil-plant systems poses a severe threat to food safety and public health. This study demonstrates that foliar application of nano-selenium fertilizer effectively reduces ARG abundance in the soil, phyllosphere, and rice grains. We found that nano-selenium functions not by direct bactericidal action but by beneficially reshaping the microbial communities in both the leaves and soil, thereby suppressing the pathways for ARG transmission. Our findings provide a novel and sustainable strategy to mitigate antibiotic resistance in agricultural ecosystems, potentially reducing the risk of these genes entering the human food chain via rice.

## INTRODUCTION

The widespread application of antibiotics in livestock production has accelerated the emergence and proliferation of antibiotic-resistant bacteria, leading to a surge in difficult-to-treat infections and increased mortality rates (1). A key driver of this trend is the proliferation of antibiotic resistance genes (ARGs)—mobile genetic elements that enable bacteria to survive antibiotic exposure and spread rapidly among microbial populations via horizontal gene transfer (HGT) (2). Antibiotic resistance genes conferring resistance to tetracyclines and sulfonamides have been identified in the phyllosphere and rhizosphere microbiomes of common crops in China, including lettuce, corn, and carrot (3, 4). Tetracycline resistance genes such as *tetM* and *tetX* have been detected in agricultural soils across 30 provinces in China, with relative abundances ranging from 10^−7^ to 10^−2^ gene copies per 16S rRNA gene copy, reflecting a 15-fold increase since the 20th century (5, 6). Moreover, paddy fields, one of the largest agricultural land systems globally, become significant reservoirs for the accumulation and dissemination of ARGs due to the prolonged application of manure and organic fertilizers (7, 8). Zhang et al. (9) demonstrated the mobilization of several ARGs—such as *aadA1*, *bla1*, and *catA1*—from paddy soil microbiomes to aquatic microbial communities, illustrating the potential for cross-habitat HGT. Once transferred to pathogenic bacteria, these ARGs can compromise antibiotic efficacy, impede the control of infectious outbreaks, and pose severe threats to public health (10, 11). It is therefore essential to mitigate the dissemination of ARGs across agricultural soil-plant systems, thereby reducing the environmental burden of antibiotic resistance and safeguarding ecosystem and human health.

Developing targeted, eco-compatible strategies to curb the proliferation of ARGs within key food systems is paramount. Selenium (Se) emerges as a promising agricultural candidate due to its recognized benefits for plant development, yield enhancement, stress resilience, and human nutrition (12). Selenium can alter the composition of microbial communities by affecting the growth and metabolism of specific microbes (13), thereby influencing the abundance and distribution of ARG hosts in the environment (14). To harness this regulatory potential with precision and minimal ecological impact, nanotechnology provides a pivotal platform. For instance, the valorization of agricultural waste into functional nanocarriers, such as nanocellulose derived from livestock manure, exemplifies a circular economy approach to substrate development (15). Similarly, innovations in energy-efficient, portable fabrication techniques, including battery-powered pressurized spinning, provide adaptable platforms for producing tailored micro- and nano-structured vehicles (16). Compared to silver or copper nanoparticles, Se nanoparticles (SeNPs) exhibit a higher biocompatibility and pose a lower direct toxicity risk to microbes (17). More critically, SeNPs can specifically inhibit plant pathogens and other potential ARG hosts by irreversibly disrupting cellular respiration, reducing dehydrogenase activity, and compromising membrane integrity (18, 19). Chen et al. (20) found that SeNPs achieved a 50% removal of tetracycline ARGs during composting, which was primarily through the suppression of transposase activity and the weakening of ARG-host linkages. Surface functionalization can significantly improve their environmental behavior and safety (17). For instance, alkyl polyglucoside-stabilized selenium nanoparticles (AG-SeNPs) improve colloidal stability, thereby mitigating the uncontrolled toxicity associated with nanoparticle aggregation and rapid ion release (21). The controlled release of Se ions from this stabilized formulation enables sustained and targeted action against bacterial hosts of ARGs, offering a potential mechanism for attenuating ARGs (22). Therefore, AG-SeNPs can be applied as a low-toxicity and effective technology for mitigating ARGs in agricultural settings.

The rhizosphere serves as a crucial arena for these interactions, where dynamic exchanges among plants, microbes, and soil components create hotspots for ARG transmission (23, 24). Bacteria are primary drivers of environmental behavior and the emergence of ARGs. Guo et al. (25) suggested that rhizosphere bacteria, including genera *Lactobacillus*, *Facklamia*, and *Devosia*, could facilitate the transfer of tetracycline resistance genes and mobile genetic elements (MGEs), thereby contributing to the regulation of ARG dissemination in the environment. Meanwhile, phyllosphere bacteria can produce natural antibiotics or other antimicrobial substances that inhibit pathogens on the surfaces of plant leaves, reducing the need for antibiotic use and thereby decreasing the selection pressure for ARGs that arises from antibiotic usage. Zhang et al. (26) demonstrated that biologically synthesized nano-selenium fertilizers reduced the expression of ARGs in broiler manure by inhibiting bacterial selenocompound metabolism and chemotaxis pathways, particularly in members of the phyla Bacteroidetes and Firmicutes. However, the processes governing the colonization and dispersal of ARG-carrying bacteria from soil to the rice plant under the influence of AG-SeNPs are still largely unknown.

The dynamics of ARGs are mediated by key environmental factors, including soil pH, electrical conductivity (EC), and organic matter (OM), which collectively shape microbial community structure and metabolic functions (27, 28). Shao et al. (29) reported that lower soil pH could diminish the abundance of typical ARG hosts such as Firmicutes, thereby limiting HGT. Li et al. (30) demonstrated that increased OM enhanced microbial competition and restrained the proliferation of ARG-carrying bacteria, ultimately constraining ARG dissemination. However, it remains unclear how AG-SeNPs interact with these established environmental drivers to reduce ARG prevalence through the regulation of rhizosphere and phyllosphere microbiomes. There is a pressing need to translate mechanistic insights from controlled experiments into actionable strategies that can be integrated into field-applicable and management-oriented strategies to curb the environmental dissemination of ARGs.

This study aimed to evaluate the efficacy of AG-SeNPs in mitigating ARGs within a paddy ecosystem, encompassing rhizosphere soil, phyllosphere, and rice grains. Six experimental treatments with and without AG-SeNP application were established to address the following objectives: (i) to quantify and profile the distribution of bacteria-associated ARGs across compartments; (ii) to characterize AG-SeNP-induced microbial community restructuring at the species level and identify key taxa and potential ARG hosts; and (iii) to uncover the mechanisms by which AG-SeNPs attenuate ARGs through microbial assembly and functional shifts based on functional prediction, random forest modeling, null model analysis, and structural equation modeling (SEM). By systematically delineating the interactions among AG-SeNPs, environmental factors, keystone taxa, and ARGs, this work provides a mechanistic basis for translating experimental findings into field-applicable strategies and retaining ecological compatibility with existing crop production systems.

## MATERIALS AND METHODS

### Preparation and characterization of AG-SeNPs

Sodium selenite (Na_2_SeO_3_), ascorbic acid, and alkyl polyglucoside were obtained from Sigma-Aldrich (St. Louis, MO, USA). AG-SeNPs were synthesized using a chemical reduction technique, referring to Sentkowska and Pyrzynska (31). Briefly, Na_2_SeO_3_ and ascorbic acid were dissolved in deionized water and mixed under continuous stirring. Alkyl polyglucoside solution (as a stabilizing agent) was added dropwise to the reaction mixture, and the reaction was allowed to proceed for 2 h at 25°C under ambient light. The AG-SeNPs were centrifuged and washed with deionized water to remove unreacted precursors, resulting in particles with a size of 44.83 ± 0.43 nm. Details on the microscopic morphology and particle size distribution of AG-SeNPs are shown in Fig. S1. The prepared AG-SeNPs were then diluted 500-fold with ultrapure water for the experiment.

### Experimental design

The pot experiments were conducted under field conditions to investigate the effects of AG-SeNPs on the abundance and distribution of ARGs in the paddy-soil ecosystem. *Wuyou-4*, a medium-late maturing rice cultivar (*Oryza sativa* L.) commonly grown in China, was used in this study. The paddy soil used in this experiment was collected from an agricultural field (41° 31' 9.24" N, 123° 22' 28.57" E) that has been subjected to long-term rice cultivation under an organic and inorganic fertilization for over 10 years. Before soil sampling, the field received regular applications of composted swine manure, and the collected soil was classified as brown soil with the following properties: pH 7.07, OM 12.41 g kg^−1^, EC 220.50 μS cm^−1^, available phosphorus (AP) 46.93 mg kg^−1^, available potassium (AK) 176.94 mg kg^−1^, available nitrogen (AN) 35.53 mg kg^−1^, and total Se content 170.99 μg kg^−1^.

Plastic pots (18 cm length × 18 cm width × 41 cm height) were filled with 5 kg of the air-dried soil, which was then flooded with water to a depth of 3–4 cm for the experiment. Six treatments were arranged in a randomized complete block design with three replicates each (Table 1). For each growth stage (tillering, grain-filling, and maturity), independent sets of pots were destructively sampled to collect soil and plant samples, ensuring that three biological replicates were obtained per treatment at each stage. The compound fertilizer (N:P_2_O_5_:K_2_O = 22:11:11) was applied at a rate of 500 kg ha^−1^, with nitrogen (N) and phosphorus (P_2_O_5_), providing approximately 110 kg N ha^−1^, 55 kg P_2_O_5_ ha^−1^, and 55 kg K_2_O ha^−1^, respectively. The biofertilizer consisted of composted swine manure. Mature seedlings (at three-leaf stage) were transplanted at three points per pot and three plants per hole. Both compound fertilizer and biofertilizer were added 1 week prior to transplantation. AG-SeNPs and Na_2_SeO_3_ solutions were applied as foliar sprays during the booting and grain-filling stages of rice growth, at which time, glutelin proteins effectively absorb Se (32).

**TABLE 1:** Fertilizer amount applied in various treatments

Treatment group	Se fertilizer (g ha^−1^ y^−1^)	Biofertilizer (t ha^−1^ y^−1^)	NPK fertilizer (t ha^−1^ y^−1^)
No Se fertilizer (NF)	0	0	0.5
Biofertilizer (BF)	0	15	0.5
Na_2_SeO_3_ (SS)	60^*a*^	15	0.5
Nano-selenium 1 (NS1)	15^*b*^	15	0.5
Nano-selenium 2 (NS2)	30^*b*^	15	0.5
Nano-selenium 3 (NS3)	60^*b*^	15	0.5

^*a*^ Presented Na_2_SeO_3_.

^*b*^ Presented AG-SeNPs.

### Sample collection of soil, leaf, and rice grain

In order to determine Se concentrations, rhizosphere soil samples were collected during the grain-filling and maturity stages by gently shaking off loosely adhering soil from the roots, followed by collecting the soil tightly attached to the root surface using sterilized tools to prevent cross-contamination between samples. At the maturity stage, phyllosphere samples were obtained simultaneously by gently swabbing the surface of fully expanded leaves using individually packaged sterile cotton swabs, with a new swab employed for each leaf and operators wearing disposable sterile gloves that were changed between different samples. Rice grains were thoroughly washed, dehusked, and sieved through a 0.15 mm mesh for Se analysis. All equipment was cleaned and wiped with 75% ethanol between samples, and grains from different plants were processed separately in a dedicated hood to minimize contamination. Additionally, all samples collected were immediately placed in sterile containers, flash-frozen, and stored at −80°C for ARG quantification to ensure nucleic acid stability and prevent microbial cross-contamination during storage.

### Determination of the physicochemical properties of soil and rice grains

#### Soil properties

The soil pH and EC were measured using a pH meter (INESA Scientific Instrument, Shanghai, China) and a conductivity meter (INESA Scientific Instrument, Shanghai, China), respectively. Available phosphorus, AK, and AN were determined following the methods described by Dong et al. (33) and Yan et al. (34). The OM content of the soil samples was assessed by measuring the loss on ignition at 550°C for 6 h, according to the method described by Zhang et al. (35). The Se concentrations were obtained according to the China Soil Environmental Quality Standards GB15618-2018 and analyzed through inductively coupled plasma mass spectrometry (ICP-MS/MS, NexION 300X, Cary, NC, USA) (36).

#### Se content in rice grains

The total Se content in polished rice was determined in accordance with the national standard method (GB5009.93-2017: National Standard for Food Safety Determination of Se in Foods) and was further analyzed by ICP-MS/MS (37).

### ARG quantification

#### DNA extraction

The total DNA of rhizosphere bacteria was extracted using the PowerSoil DNA Isolation Kit (Qiagen, Hilden, Germany), and bacterial DNA from phyllosphere samples and rice grains was extracted using the Plant DNA Extraction Kit (Qiagen, Hilden, Germany) according to the manufacturer’s instructions.

#### ARG analysis

Referring to our previous study, a customized gene chip targeting 20 tetracycline resistance genes (T-ARGs: *tet32*, *tet36*, *tet44*, *tetB*, *tetC*, *tetE*, *tetH*, *tetJ*, *tetM*, *tetO*, *tetQ*, *tetR*, *tetS*, *tetT*, *tetW*, *tetX*, *tetbP*, *tetGF*, *tetPA*, and *tetPB*), 10 quinolone, 2 aminoglycoside-resistance genes (QA-ARGs: *aac(6')-Ib*, *oqxA*, *qnrA*, *qnrB4*, *qnrB-bob_resign*, *qnrB46,47,48*, *qnrD*, *qnrS1_S3_S5*, *qnrS2*, *qnrVC1_VC3_VC6*, and *qnrVC4_VC5_VC7*), and 2 MGEs (*int-I1* and *int-I2*) were employed to quantify the abundance of these genes in the extracted DNA samples (38). The target genes were quantified with a high-throughput quantitative PCR system (WaferGen SmartChip) by Differential Gene Technology Co., Ltd. (Anhui, China), following standardized protocols. Specific primes for the aforementioned are listed in Table S1.

### Microbial community analysis

#### 16S rRNA amplicon sequencing

To achieve improved taxonomic resolution at the species level, the full-length bacterial 16S rRNA gene was amplified and sequenced. PCR amplification was performed using the primer pair 27F (5′-AGRGTTYGATYMTGGCTCAG-3′) and 1492R (5′-RGYTACCTTGTTACGACTT-3′). The resulting amplicons were sequenced on a PacBio Sequel II platform (Pacific Biosciences, Menlo Park, CA, USA), which provides long-read sequencing capable of spanning the entire ~1,500 bp 16S rRNA gene. After quality filtering and denoising, an average of 37,102 ± 2,747 and 33,467 ± 2,656 high-quality full-length 16S rRNA gene sequences per sample were obtained for the rhizosphere soil and phyllosphere communities, respectively. Shannon diversity–based rarefaction curves for all samples approached a plateau with increasing sequencing depth. Detailed per-sample sequencing statistics and rarefaction curves are provided in Table S2 and Fig. S2, respectively.

#### Bioinformatics analysis

Raw sequencing reads were processed using the QIIME2 pipeline (39). Quality filtering, denoising, and chimera removal were performed using the DADA2 plugin. Amplicon sequence variants (ASVs) were generated and taxonomically classified using a pre-trained Naive Bayes classifier based on the SILVA 138 99% OTUs reference database (40).

#### Data analysis

Statistical analyses were conducted using R software (version 4.2.3). A one-way analysis of variance (ANOVA) followed by Tukey’s Honestly Significant Difference (HSD) was applied to compare the means of ARG abundance and alpha diversity indices among different treatments. Hierarchical clustering with Ward’s minimum variance method was utilized to group samples based on their similarity in ARG profiles for cluster analysis. Co-occurrence networks were constructed based on Pearson correlations and performed using the Gephi software (version 0.10.1) to investigate correlations between ARGs and bacterial species. Redundancy analysis (RDA) was performed with the “vegan” package in R to quantify the contributions of environmental factors, MGEs, and microbial communities to the variation in ARG abundance.

Mantel tests were conducted using the vegan package in R with 9,999 permutations to assess the correlations between microbial community structures and ARG/MGE profiles. Pairwise Pearson or Spearman correlations were computed and visualized using the ggcor/linkET workflow, where correlation matrices were presented together with significant Mantel associations (*P* < 0.05). Because this analysis aimed to explore the association patterns rather than perform formal hypothesis testing on each individual pair, *P*-values for pairwise correlations were not adjusted. PICRUSt2 was applied to predict putative functional profiles from full-length 16S rRNA gene sequences (41), leveraging the enhanced phylogenetic resolution of long reads to improve prediction accuracy. In this study, PICRUSt2 outputs were used to identify broad functional tendencies and were interpreted jointly with directly measured data—ARG and MGE abundances and microbial community composition—ensuring that predicted functions served as a complementary layer in an integrated analytical framework. Random forest modeling was conducted using the “randomForest” package to identify key microbial taxa and environmental variables predictive of ARG and MGE abundance (42, 43). To infer community assembly processes, a null model framework was applied following the protocol of Sun et al. (44), implemented via the “picante” package. The beta nearest taxon index (βNTI) and Bray-Curtis-based Raup-Crick (RCbray) were calculated to quantify phylogenetic and taxonomic turnover. Specifically, βNTI values greater than +2 or less than –2 were interpreted as indicative of deterministic processes, with βNTI>+2 reflecting heterogeneous selection and βNTI<–2 indicating homogeneous selection. |βNTI|<2 represents that stochastic processes dominate community assembly. RCbray <0.95 represents homogenizing dispersal, and RCbray >0.95 indicates dispersal limitation. If |βNTI|<2 and |RCbray|<0.95, community assembly is considered undominated, primarily driven by weak selection, weak dispersal, diversification, and/or drift (44). For a more comprehensive insight into the interplay among potential predictors and the dynamics of ARGs and MGEs, structural equation models (SEM) were conducted using the “piecewiseSEM v2.1.2” package. Additionally, variance partitioning analysis (VPA) was carried out using the “vegan” package in R to quantify the contributions of various factors of variation to ARG abundance.

### Statistical analysis

Statistical differences among treatment groups were evaluated using ANOVA in R (version 4.5.0). Upon detecting a significant effect, Duncan’s multiple range test was performed for post-hoc comparisons using the “agricolae” package. Pearson correlation coefficients were calculated to assess relationships among variables. Graphs and supplementary statistical analyses were generated in OriginLab 2022 and R.

## RESULTS

### Se levels in rhizosphere, phyllosphere, and rice grains

The total Se content in the soil, leaves, and rice grains across different growth stages is presented in Fig. S3. Foliar application of both Na_2_SeO_3_ and AG-SeNP solutions significantly increased Se content in rice leaves and polished rice, while no significant change was observed in soil Se levels (*P* > 0.05). The Se levels in leaves increased as the concentration of AG-SeNPs enhanced (*P* < 0.05), and the Se content in leaves in NS3 reached 672.40 ± 53.02 µg kg^−1^ and 1,600.61 ± 54.33 µg kg^−1^ at the grain-filling and maturity stage, respectively, following the application of 60 g ha^−1^ AG-SeNPs. The Se content in rice grains initially increased and subsequently decreased as the AG-SeNP concentration ranged from 15 g ha^−1^ (NS1) to 60 g ha^−1^ (NS3). The highest Se level in rice grains was observed with the application of 30 g ha^−1^ AG-SeNPs (NS2), achieving 413.02 ± 31.32 µg kg^−1^, which represented a 2.1-fold increase compared to the NF treatment. Although the Se content in rice grains under the SS treatment at maturity was not significantly different from that under the NS2 group (*P* > 0.05), the amount of Na_2_SeO_3_ solution in the SS was double that of the AG-SeNP solution in NS2. The application of 30 g ha^−1^ AG-SeNPs is thereby considered the optimal strategy for Se enrichment in rice in this study.

### Distribution patterns of ARGs and MGEs in bacterial communities of rhizosphere, phyllosphere, and rice grains

**Fig 1:**
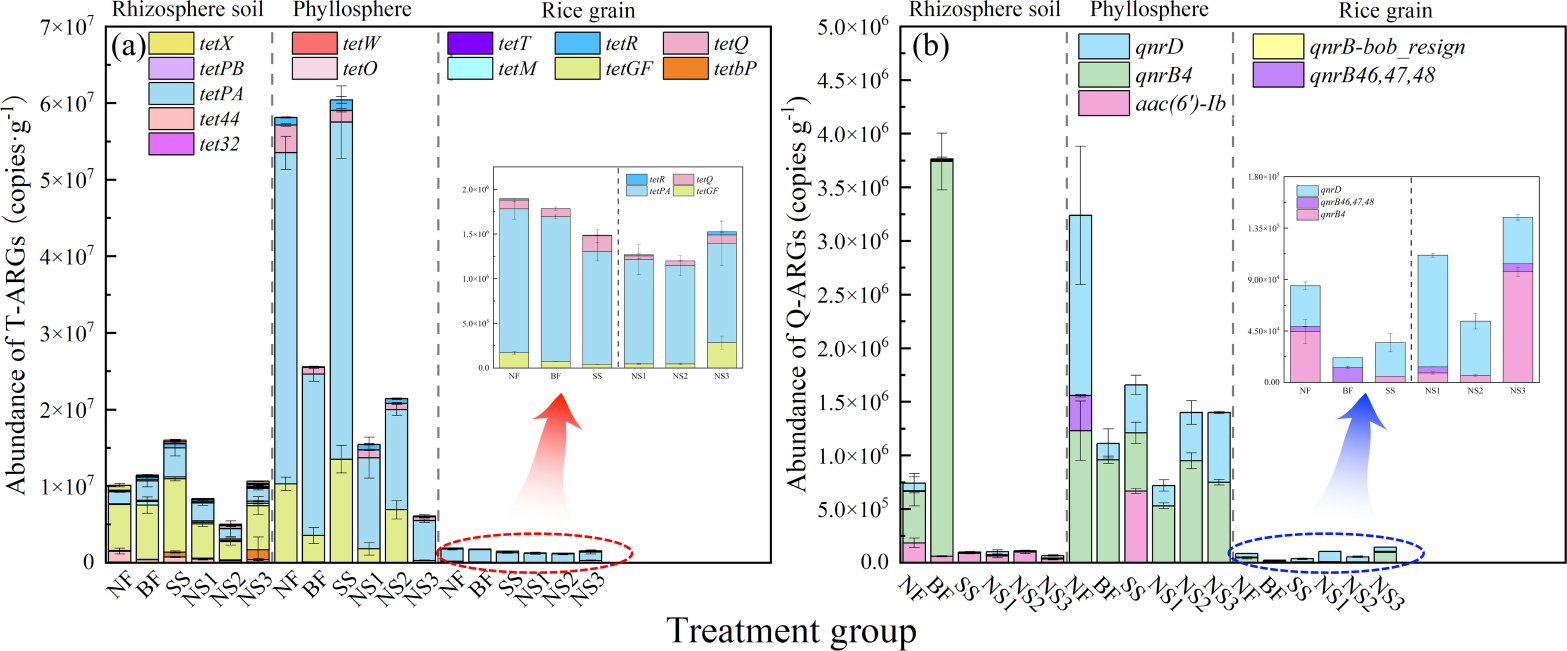
Copy numbers of (**a**) T-ARGs and (**b**) QA-ARGs among bacteria in rhizosphere soil, phyllosphere, and rice grains.

Marked variations in the abundance and composition of ARGs were observed among the rhizosphere, phyllosphere, and rice grains under different treatments, as illustrated in Fig. 1. The ARG copy numbers in bacteria followed the trend: phyllosphere > rhizosphere > rice grains. T-ARGs (*tetPA* and *tetGF*) exhibited notable copy numbers across the paddy-soil ecosystem. In the rhizosphere, foliar application of AG-SeNPs significantly reduced the copy numbers of T-ARGs (*tetX*, *tetGF*, and *tet44*) and QA-ARGs (*qnrB4*, *qnrD*, and *aac(6’)-Ib*) compared to the control group (NF: 1.08 × 10^7^ copies g^−1^), with the total copy numbers of ARGs decreasing by 21.81% (NS1), 52.60% (NS2), and 1.20% (NS3), respectively. In the phyllosphere, the total ARG copy numbers exhibited a unimodal pattern with AG-SeNP concentration, increasing from NS1 (15 g ha⁻^1^) to NS2 (30 g ha⁻^1^) and then decreasing at NS3 (60 g ha⁻^1^), with the lowest value (1.62 × 10^7^ copies g^−1^) observed in NS1. An apparent reduction in *tetPA*, *tetGF*, *tetQ*, *qnrB4*, and *qnrD* in the phyllosphere bacteria was observed after applying AG-SeNPs. However, the application of Na_2_SeO_3_ significantly upregulated ARGs such as *tetGF* and *aac(6’)-Ib* in phyllosphere bacteria, resulting in a total copy number of 8.56 × 10^8^ copies g^−1^ (SS). Compared to the rhizosphere and phyllosphere, the total copy numbers of ARGs in rice grain bacteria decreased by an order of magnitude. *TetPA* was identified as the dominant ARG in rice grain microbial communities, with 1.48 × 10^6^ copies g^−1^ in the NF treatment. The use of AG-SeNPs significantly reduced the copy numbers of ARGs in grain bacteria (Fig. 1a). Specifically, the application of 30 g ha^−1^ AG-SeNPs reduced T-ARGs to 1.25 × 10^6^ copies g^−1^, corresponding to reduction of a 31.91% and 23.64% relative to the NF and BF groups, respectively. However, an increase in the abundance of *qnrD* and *qnrB46, 47, 48* was observed following the application of 15 g ha⁻¹ AG-SeNPs (NS1) (Fig. 1b).

**Fig 2:**
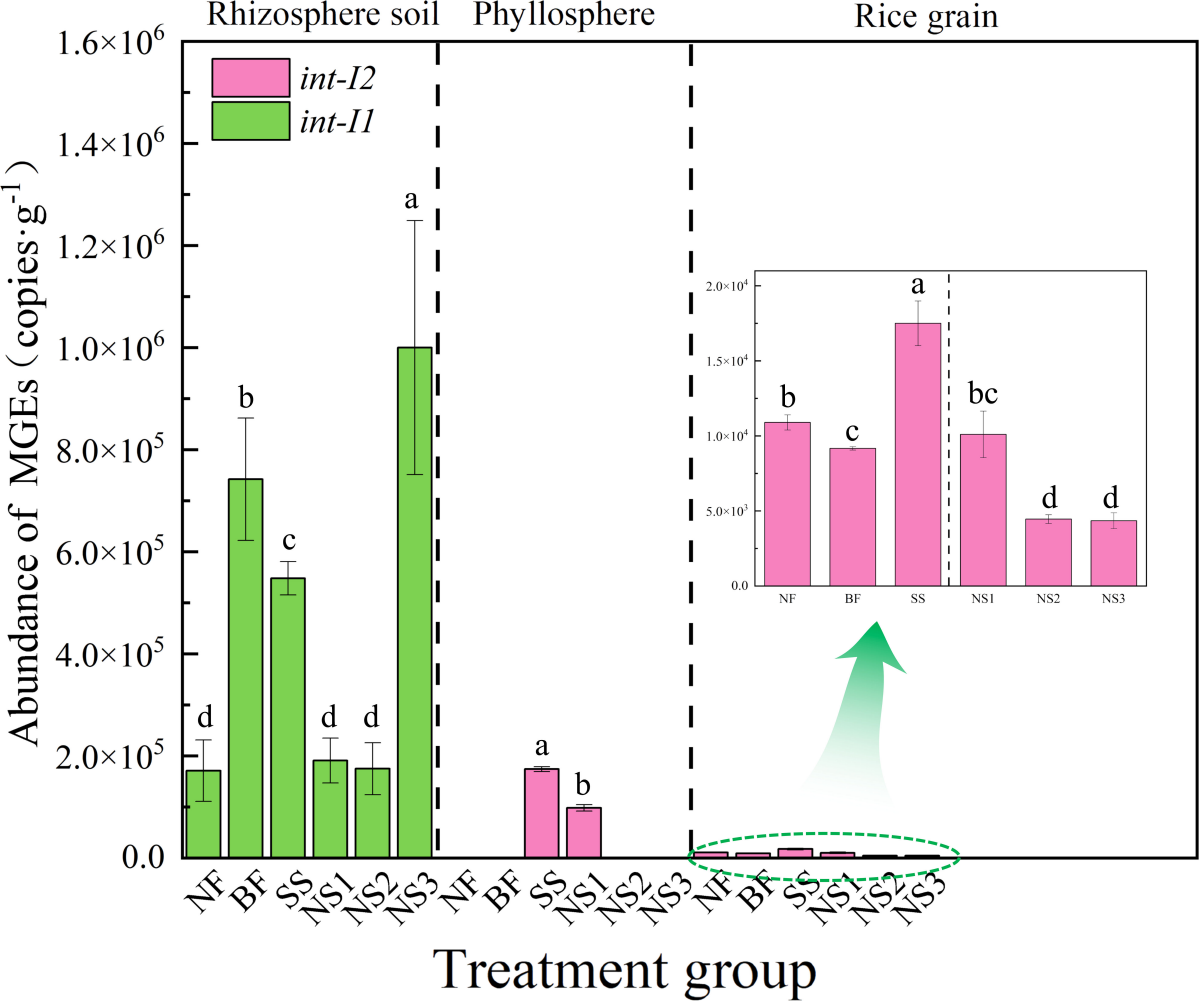
Abundance of MGEs in rhizosphere soil, phyllosphere, and rice grains. Different lowercase letters indicate significant differences among treatments (*P* < 0.05).

AG-SeNPs suppressed the abundance of MGEs, whereas Na_2_SeO_3_ exhibited the opposite trend (Fig. 2). The application of 15 and 30 g ha^−1^ AG-SeNPs did not increase *int-I1* in the soil compared to the NF group (*P* > 0.05) but reduced it by 74.24% and 76.26%, respectively, relative to the BF group. As the amount of AG-SeNPs increased to 60 g ha^−1^, *int-I1* reached 1.00 × 10^6^ copies g^−1^. *Int-I2* was detected in the bacterial communities from phyllosphere (SS and NS1 groups) and rice grains (all treatment groups). The application of 60 g ha^−1^ Na_2_SeO_3_ significantly increased *int-I2* in rice grains, whereas 30 and 60 g ha^−1^ AG-SeNPs reduced the abundance of *int-I2* in rice grains by 51.37% (4.46 × 10^3^ copies g^−1^) and 52.56% (4.35 × 10^3^ copies g^−1^), respectively, compared to the BF treatment.

In summary, the application of 30 g ha⁻¹ AG-SeNPs demonstrated optimal performance in reducing the abundance of both ARGs and MGEs among bacteria in the rhizosphere, phyllosphere, and rice grains, highlighting its potential as a more environmentally friendly alternative.

### Microbial community variations and their correlation with ARGs and MGEs

**Fig 3:**
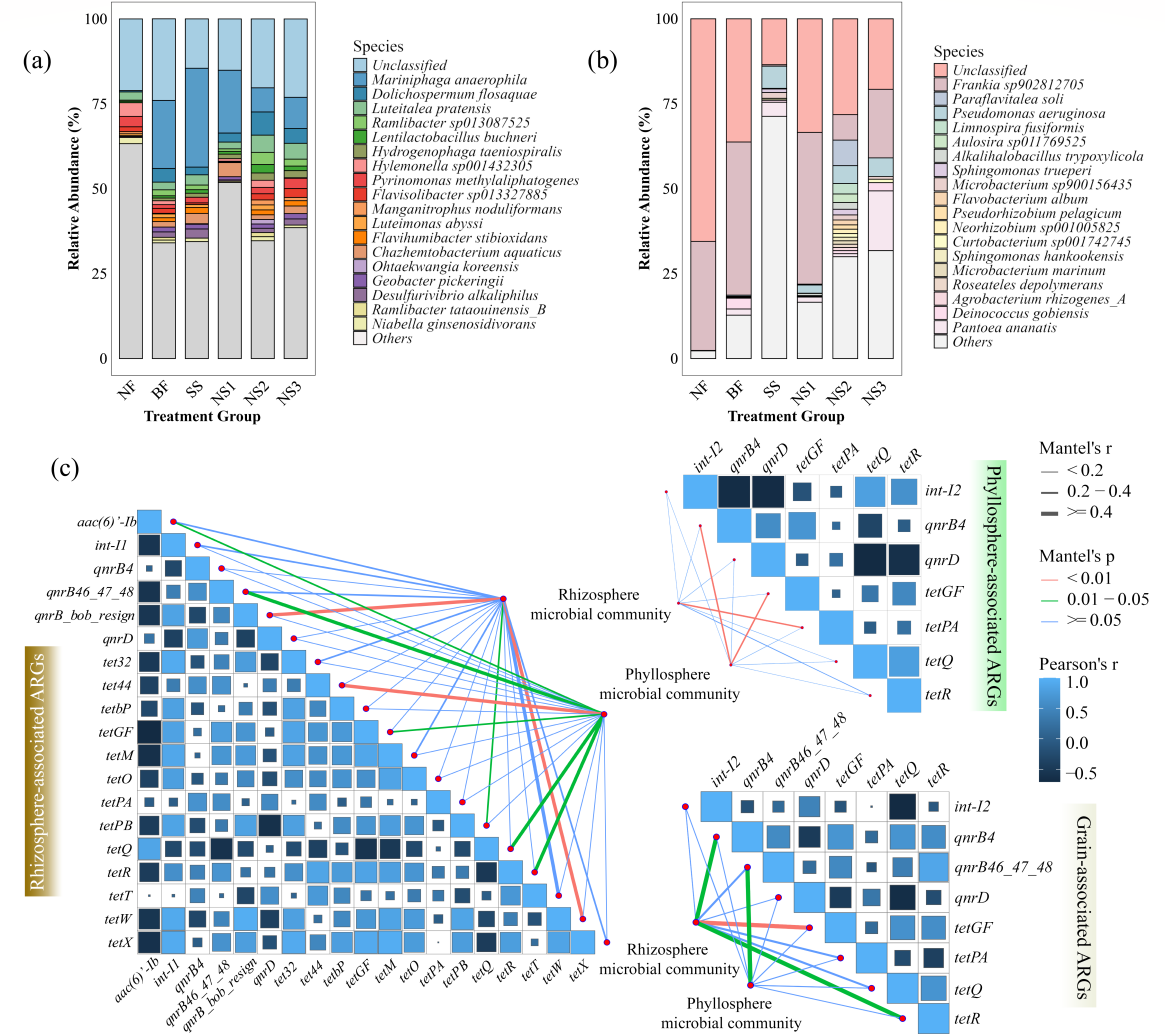
Microbial community composition at the species level in the (**a**) rhizosphere and (**b**) phyllosphere samples, along with their correlation with ARGs and MGEs in the (**c**) rhizosphere, phyllosphere, and rice grains under NS2 treatment.

Based on the ASV analysis, the bacterial community compositions of soil and phyllosphere samples at the phylum, genus, and species levels are depicted in Fig. S4 and Fig. 3. Bacteroidetes and Proteobacteria were identified as the dominant phyla in the rhizosphere soil across all treatments, accounting for 26.52% (NS2)~45.99% (NS1) and 29.14% (SS)~39.29% (NS2), respectively (Fig. S4a). Significant shifts in relative abundance of soil bacteria at the genus level were observed after applying Se solution (Fig. S4c). In comparison to the NF group, the relative abundance of *Ramlibacter*, *Mariniphaga*, *Dolichospermum*, and *Luteitalea* increased apparently in NS2 (*P* < 0.05). However, *Mariniphaga anaerophila* exhibited the highest relative abundance at 7.12%, followed by *Dolichospermum flosaquae* (6.83%), *Luteitalea pratensis* (5.08%), and *Ramlibacter sp013087525* (3.58%) (Fig. 3a).

The phyllosphere bacterial community was primarily dominated by Proteobacteria and Actinobacteria (Fig. S4b). However, the application of AG-SeNP solution (NS2 and NS3) led to a decrease in the relative abundance of these phyla, concomitant with an increase in Bacteroidetes compared to the control group (NF). This shift in community composition was further characterized by a substantial decline in *Frankia* and a corresponding increase in *Paraflavitalea* (Fig. S4d). Specifically, the relative abundance of *Frankia sp902812705* decreased from 32.11% (NF) to 7.51% (NS2). *Paraflavitalea soli* and *Pseudomonas aeruginosa*, which were nearly undetectable in NF (0.04% and 0.02%, respectively), increased to 7.51% and 5.23%, respectively, in NS2 (Fig. 3b). Furthermore, 30 g ha^−1^ AG-SeNP application (NS2) resulted in the appearance of *Limnospira fusiformis* (3.08%) and *Aulosira sp011769525* (2.51%), which were absent in the NF or BF treatments (Fig. 3b).

The mantel test analysis of soil, phyllosphere, and rice samples after AG-SeNP application is presented in Fig. 3c. The results demonstrated that ARGs among bacteria in the soil, phyllosphere, and rice were closely associated with the microbial community in the soil and phyllosphere. Soil bacteria directly influenced the relative abundance of *qnrb-bob*, *tetPB*, and *tetW. Int-I1* exhibited a negative correlation with *aac(6’)-Ib* and *qnrD* but was positively associated with *tetGF, tetW*, and *qnrb-bob_resign* (*P* < 0.05). Additionally, ARGs, such as *tet32*, *tetbP*, *tetGF*, *tetM*, and *qnrB-bob_resign*, showed positive interrelationships (*P* < 0.05). Both soil and phyllosphere bacteria contributed to variations in the abundance of *qnrB4* and *qnrB46,47,48* in leaves (Fig. 3c). In rice grains, soil microbial communities were associated with *qnrB4*, *tetPA*, and *tetQ*, and the copy number of *qnrB46,47,48* was influenced by phyllosphere bacteria. *Int-I2* was negatively linked to *qnrB4* and *qnrD* (*P* < 0.05) but positively related to *tetQ* (*P* < 0.05, r = 0.78). An increase in the copy number of *qnrD* effectively reduced the copy numbers of *tetQ* and *tetR*. Although positive correlations were observed among *tetR*, *qnrB4*, *qnrB46*,*47*,*48*, *tetGF*, and *tetQ* in rice, *int-I2* showed no significant effect on ARGs (*P* > 0.05).

### Identification of potential bacterial hosts of ARGs

**Fig 4:**
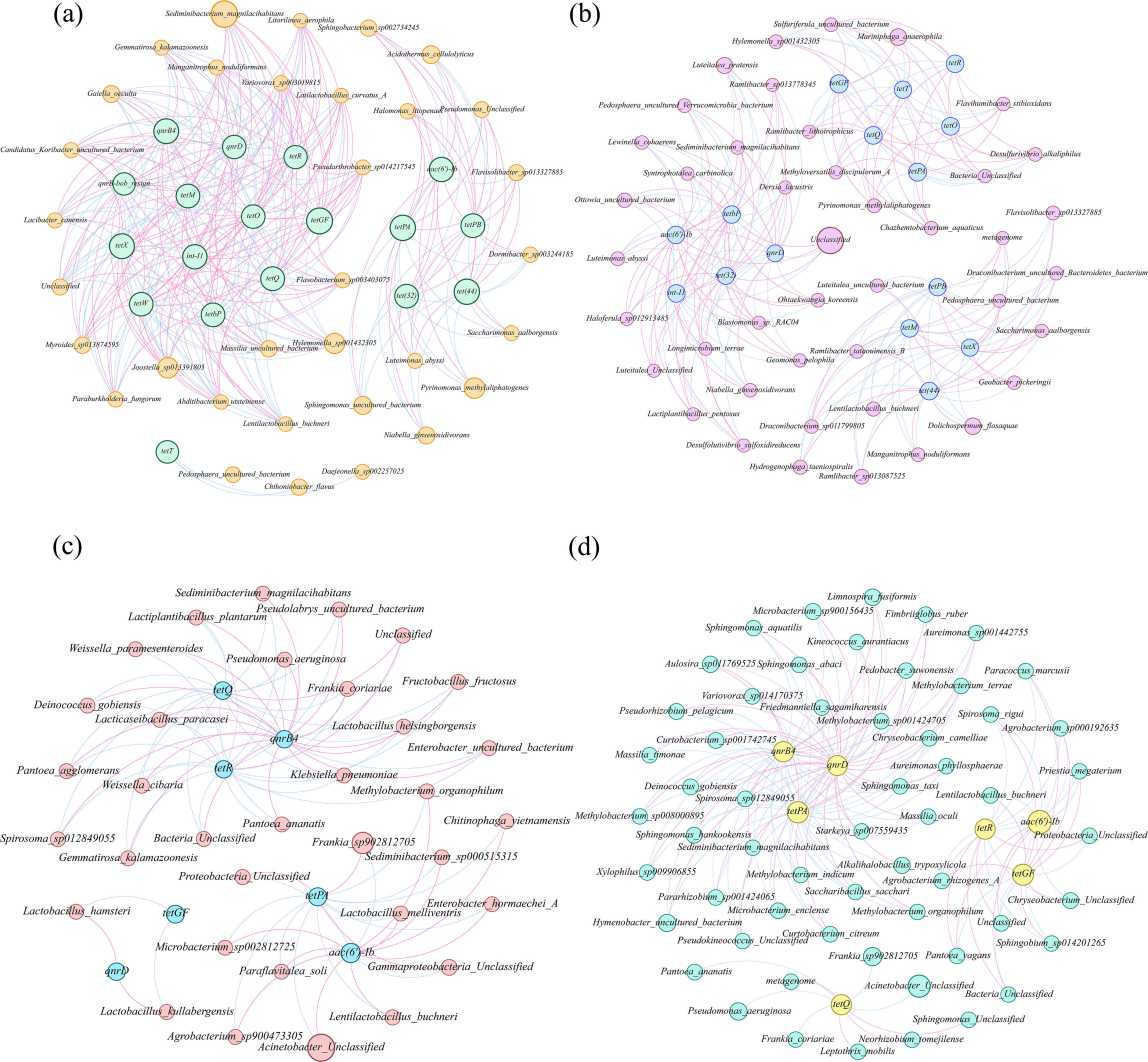
Co-occurrence networks of bacterial taxa and their associations with ARGs: (**a**) soil under NF treatment, (**b**) soil under NS2 treatment, (**c**) phyllosphere under NF treatment, and (**d**) phyllosphere under NS2 treatment. Co-occurrence edges were retained only when |r|>0.90 and *P* < 0.01 to ensure robust associations. Positive and negative correlations were visualized in Gephi as pink and blue edges, respectively. Node size was scaled according to the relative abundance of bacterial taxa or ARG copy numbers.

Due to the significant reduction in ARGs observed under NS2 treatment, a comparative analysis with the control group (NF) was performed to identify functional taxa for prevalent ARGs (Fig. 4). Network analysis based on strong correlations (r>0.9) revealed co-occurrence patterns between bacterial taxa (species level) and ARGs, facilitating the identification of potential ARG hosts. The application of AG-SeNPs altered the relationships between the microbial communities and ARGs in soil. In both NF and NS2 groups, *Lentilactobacillus buchneri* and *Hylemonella sp001432305* showed positive associations with *tetX* and *tetGF* (Fig. 4a and b). The top 19 species identified in Fig. 3a displayed strong associations with ARGs in the NS2 group (Fig. 4b). Specifically, the relative abundance of *Mariniphaga anaerophila*, *Pyrinomonas methylaliphatogenes*, *Desulfurivibrio alkaliphilus*, and *Chazhemtobacterium aquaticus* was positively correlated with *tetGF* but negatively correlated with *tetPA. Luteitalea pratensis* and *Luteimonas abyssi* were positively linked to *int-I1*. Additionally, QA-ARGs, including *qnrB4* and *qnrB-bob_resign*, were not influenced by bacteria under NS2 treatment (Fig. 4b, *P* > 0.05). However, these ARGs were positively associated with *Hylemonella sp001432305*, *Lacibacter cauensis*, and *Lentilactobacillus buchneri* in the NF group (Fig. 4a).

According to Fig. 4d, in the NF group, *Lactobacillus hamster* and *Lactobacillus kullabergensis* were the only species positively associated with *qnrD*. However, species such as *Alkalihalobacillus trypoxylicola*, *Microbacterium sp900156435*, *Pseudorhizobium pelagicum*, *Curtobacterium sp001742745*, *Sphingomonas hankookensis*, *Deinococcus gobiensis*, and *Microbacterium enclense* exhibited negative correlations with *qnrD* and *tetPA* while showing positive associations with *qnrB4* in the NS2 group (Fig. 4e). Moreover, an increase in the relative abundance of *Limnospira fusiformis* and *Aulosira sp011769525* corresponded to a decline in *qnrD*, but an increasing *tetPA* in this study. *P. aeruginosa* was positively correlated with *tetQ*, and its abundance decreased following AG-SeNPs application, which was accompanied by a reduction in the copy number of *tetQ*.

### Relationships between microbial communities and environmental factors

**Fig 5:**
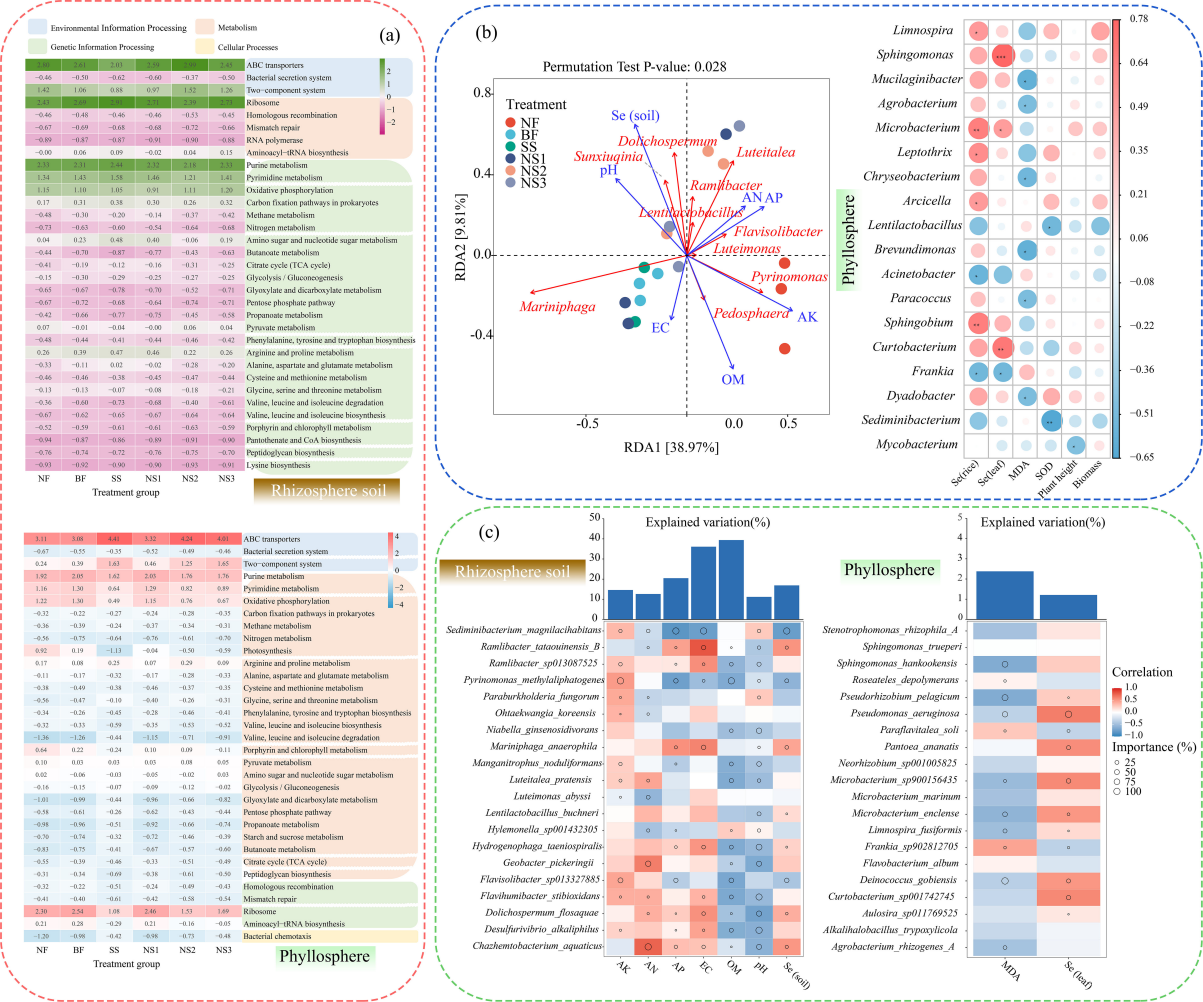
Associations between microbial community structure, predicted functions, and environmental variables: (**a**) predicted KEGG pathways of rhizosphere and phyllosphere microbiota, (**b**) environmental associations of rhizosphere and phyllosphere microbiota revealed by RDA and correlation heatmap, and (**c**) correlations and explained variation of rhizosphere and phyllosphere taxa with environmental factors based on Spearman and random forest models.

Functional prediction identified three dominant metabolic pathways in soil based on their relative abundance and pronounced treatment responses: ATP-binding cassette (ABC) transporter from environmental information processing, ribosomes from genetic information processing, and pyrimidine metabolism from metabolic processes (Fig. 5a). Compared to the NF group, the prediction of ABC transporters increased, while ribosome and purine pathways decreased under the NS2 group. The functional prediction of the phyllosphere bacteria revealed significant variations in crucial metabolic pathways, characterized by enhanced activity in two-component system and arginine and proline metabolism, alongside reduced photosynthesis and ribosome activity. These functional shifts were associated with the observed reduction in ARGs, reflecting changes driven by multiple ecological and metabolic mechanisms.

Redundancy analysis illustrated the relationship between soil environmental factors and microbial community compositions (Fig. 5b). Soil environmental factors significantly explained the variation in microbial community composition (*P* < 0.05), and the first two RDA axes accounted for 48.78% of the total variation. Among the measured environmental factors, Se concentration, pH, and OM were identified as the primary drivers influencing microbial community compositions. Selenium concentration showed a strong positive correlation with the relative abundance of *Dolichospermum* (r = 0.41, *P* < 0.05) and *Sunxiuqinia* (r = 0.52, *P* < 0.05), both of which were also positively linked to pH. Organic matter content was negatively associated with the relative abundance of *Luteitalea* (r= −0.71, *P* < 0.05) and *Ramilibacter* (r = −0.48, *P* < 0.05). As illustrated in Fig. 5b, Se levels in both rhizosphere and phyllosphere samples were positively associated with the relative abundance of *Microbacterium* (*P* < 0.05). Selenium levels in soil displayed positive correlations with the relative abundance of *Sphingobium* (*P* < 0.05), *Limnospira* (*P* < 0.05), and *Leptothrix* (*P* < 0.05), while showing negative correlations with *Acinetobacter* and *Frankia* (*P* < 0.05). The concentrations of Se in phyllosphere samples were positively correlated with *Sphingomonas* (*P* < 0.001) and negatively correlated with *Frankia* (*P* < 0.05). However, microbial community compositions could not influence the biomass of the rice plants (*P* > 0.05).

The random forest model identifies key environmental drivers associated with rhizosphere and phyllosphere microbial taxa (Fig. 5c). The explained variation of environmental variables ranged from 11.20% to 39.41%, with the highest values observed for OM (39.41%). The relative abundances of *Luteitalea pratensis, Hydrogenophaga taeniospiralis*, *Pyrinomonas methylaliphatogenes*, and *Flavisolibacter sp013327885* were significantly negatively correlated with OM. Among these, *Pyrinomonas methylaliphatogenes* exhibited the highest predictive importance for OM and also showed a strong association with AK, with a predictive importance of 100% for both variables. *Ramlibacter tataouinensis B* was positively correlated with EC (r = 0.71, *P* < 0.01), while *Chazhemtobacterium aquaticus* showed a positive correlation with AN (r = 0.70) and a negative correlation with pH (r = −0.31). *Pyrinomonas methylaliphatogenes* and *Sediminibacterium magnilacihabitans* were both negatively correlated with soil Se concentrations, and the latter also had high predictive importance for EC and AP. Moreover, *Pseudomonas aeruginosa* in the phyllosphere was negatively correlated with MDA (r = −0.64) and positively correlated with Se (r = 0.27), with predictive importance of 100% for Se and 60.95% for MDA. *Deinococcus gobiensis* exhibited a predictive importance of 100% for MDA.

**Fig 6:**
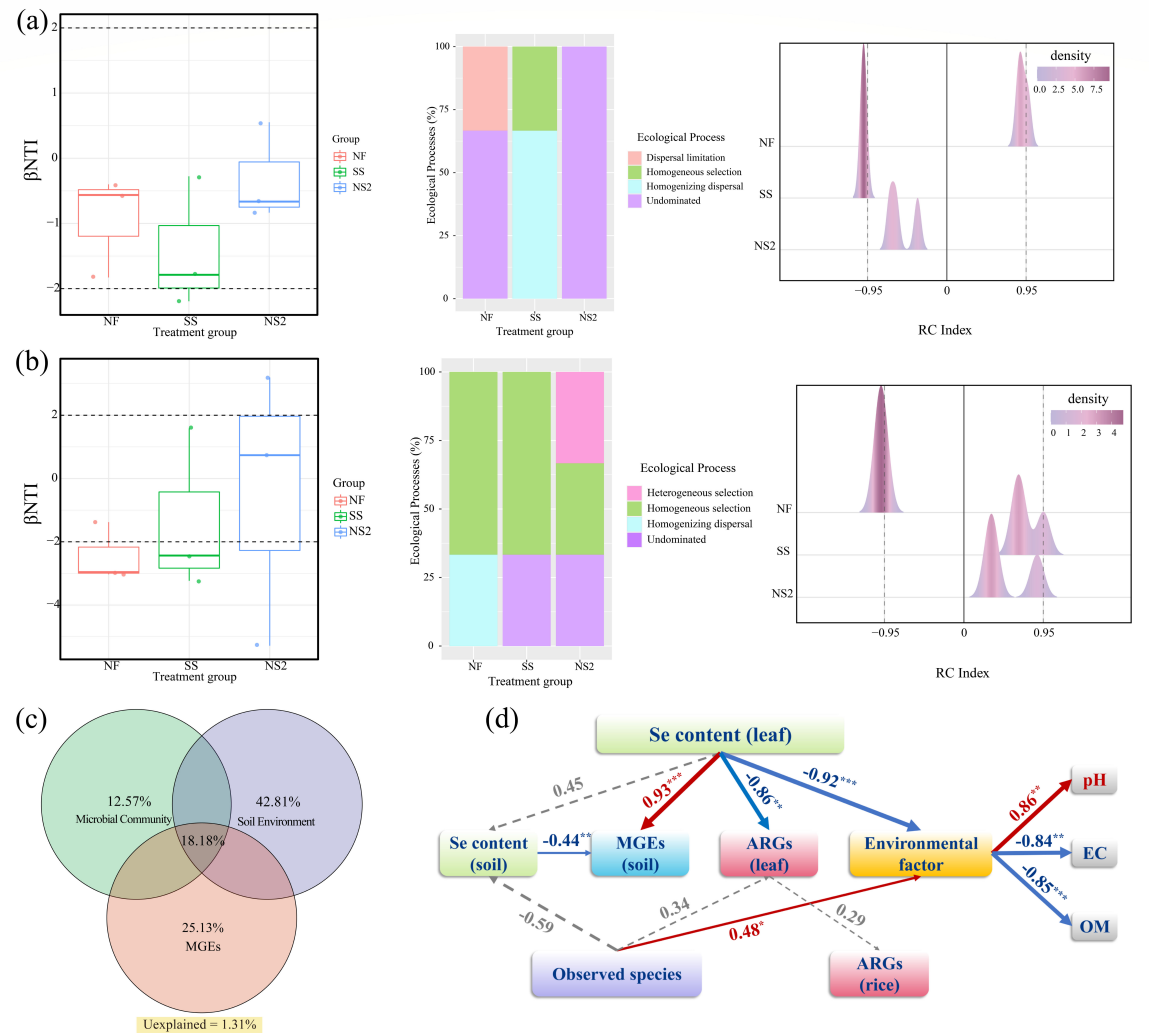
Ecological processes and regulatory pathways underlying ARG attenuation: null model analysis of microbial community assembly in (**a**) rhizosphere and (**b**) phyllosphere, (**c**) VPA analysis, and (**d**) SEM analysis; solid red lines denote positive correlations, solid blue lines denote negative correlations, and dashed gray lines denote the absence of a significant correlation.

The contributions of deterministic and stochastic processes to microbial community assembly were assessed under different treatments (Fig. 6a and b). In rhizosphere bacterial communities (Fig. 6a), βNTI values across all treatments fell within the range of –2 to +2, corresponding to stochastic assembly patterns. Ecological process partitioning showed that homogenizing dispersal dominated in the SS group, while community assembly in the NF group was entirely driven by stochastic processes, including dispersal limitation and drift. All pairwise RC values in NS2 ranged between –0.95 and 0 and were classified within the drift.

In phyllosphere microbes (Fig. 6b), deterministic processes were predominant across treatments, with deterministic contributions reaching 66.67% in all groups. Although βNTI values showed no significant differences among treatments, NS2 exhibited a higher proportion of heterogeneous selection (33.33%). RC values in NF were primarily between –1 and –0.95, corresponding to homogenizing dispersal, whereas RC values in SS and NS2 were distributed between 0 and 0.95, consistent with ecological drift as the dominant stochastic process.

According to the VPA results (Fig. 6c), the antibiotic resistome was primarily governed by soil physicochemical properties, bacterial communities, and MGEs. Environmental variables had the most significant impact, accounting for 42.81% of the ARG variation, while the combined (shared) contribution of these three factors accounted for 18.18% of the total explained variance in ARG distribution. Among these environmental variables, selenium emerged as a key driver mediating the linkage between soil and phyllosphere systems. Structural equation modeling identified foliar selenium content—elevated by AG-SeNP application—as a key factor mitigating ARGs (Fig. 6d). Higher leaf Se concentrations were associated with increased soil Se content and altered environmental parameters (OM, EC, and pH), which further affected the abundance of ARGs and MGEs in soil. Although Se exhibited an overall mitigating trend on leaf ARGs, the relationship between Se content in leaves and soil ARGs was non-linear (*P* > 0.05).

## DISCUSSION

### Mechanisms of Se enrichment in rice grains

The superior efficacy of AG-SeNPs compared with Na_2_SeO_3_ stems from their distinct uptake and translocation pathways. Sodium selenite, primarily absorbed by plant roots via phosphate transport channels in a metabolically active process, supplies Se in ionic forms (45). Once absorbed, selenite is reduced to selenide and incorporated into selenocysteine via the Se assimilation pathway. In contrast, AG-SeNPs are directly absorbed by plant leaves via stomata, adhere to cell membranes, and facilitate intracellular transport independent of root-mediated pathways (37). The application of AG-SeNPs minimizes direct Se accumulation in soils, and negligible changes of Se content in soils are thereby observed across treatments. Their nanoparticulate nature ensures better bioavailability, larger surface area, and enhanced interaction with cellular transport systems (45, 46). As a result, a 30 g ha^−1^ AG-SeNP treatment achieved Se enrichment in rice grains comparable to a 60 g ha^−1^ Na_2_SeO_3_ treatment, demonstrating the superior resource efficiency of AG-SeNPs.

Following the AG-SeNP application, Se levels in rice grains ranged from 206.62 to 413.02 µg kg^−1^, indicating its potential for Se fortification. The observed dose-dependent increase in leaf Se content aligns with established physiological roles of selenium in plants. Selenium serves as a catalytic center for glutathione peroxidase and chloroplast-associated enzymes, scavenging free radicals and shielding the photosynthetic apparatus from oxidative damage (47, 48). This dual role enhances chlorophyll synthesis, promotes plant growth, and facilitates efficient Se accumulation (37, 38). This unique absorption mechanism accelerates Se uptake and ensures more efficient translocation throughout the plant (49). The enhanced cellular absorption efficiency of SeNPs at moderate concentrations facilitates the optimal translocation of Se to grains (50). However, excessive AG-SeNP application (60 g ha^−1^) led to reduced grain Se content, suggesting phytotoxic effects. At higher concentrations, physiological stress may impair the functionality of Se transport proteins, redirecting Se to non-grain tissues. Additionally, the observed decrease in the availability of key nutrients such as nitrogen, phosphorus, and potassium under high Se treatment could further limit the plant’s capacity for efficient Se assimilation and translocation to grains. Applying AG-SeNPs also stimulated the chemotactic response of *Sphingomonas* and the proliferation of *Pseudomonas*, both of which are associated with enhanced Se enrichment process and organic acid secretion (51). These plant-microbe interactions potentially accelerate Se uptake and internal redistribution.

In summary, the application of 30 g ha^−1^ AG-SeNPs integrates material efficiency, physiological compatibility, and microbial facilitation, offering a dual benefit of reduced Se input and effective grain biofortification.

### Effects of AG-SeNP application on ARGs

A consistently high abundance of ARGs was detected in phyllosphere bacterial communities across treatments, including the NF treatment, reflecting the legacy effect of long-term organic fertilization that enriches ARG-harboring microbes in soil and facilitates their aerial dispersal to leaves (25, 52). The phyllosphere’s broad surface area and exposure to manure-derived particulates create a favorable microenvironment for ARG acquisition via dry deposition, contact transfer, and microbial colonization (53). The spread of ARGs among phyllosphere bacteria is thereby not easily replicated in soil or rice tissues, and the abundance of ARGs in the phyllosphere bacteria is markedly higher than that in the soil or rice samples (Fig. 3a and b).

Selenium application exerted multifaceted influences on ARG profiles. Most detected T-ARGs (excluding *tetR*) function through efflux pumps and enzymatic modification. In this study, AG-SeNPs were found to reduce the abundance of efflux pump genes (*tetGF* and *tetQ*) in the soil, phyllosphere, and rice tissues (Fig. 1a), likely by lowering oxidative stress and thereby reducing ARG selection pressure. Owing to their great bioactivity and nanoparticle-mediated slow release (54), AG-SeNPs enhance selenium bioavailability while minimizing cellular stress responses, thereby avoiding the activation of compensatory resistance-related gene expression (55–57). In contrast, Na_2_SeO_3_ upregulated *tetGF* and *tetPA* (Fig. 1a), potentially due to its ionic form inducing stronger oxidative stress and enriching Se-tolerant bacteria often possessing co-resistance to antibiotics (58).

Integrase genes can promote HGT of ARGs among bacteria (59, 60). The integrase gene *int-I1*, predominantly detected in rhizosphere soil, was significantly reduced following Ag-SeNP application. As *int-I1* facilitates the dissemination of *tetGF* and *tetW*, its decline suggests a suppression of soil-to-plant ARG transfer pathways (60). Consequently, the observed resurgence of *tetGF* in NS3 is directly linked to an increase in *int-I1* abundance, which restored its role in facilitating gene transfer.

The integrase gene *int-I2* was mainly detected in bacteria from the phyllosphere (SS and NS1 treatments) and rice grains (Fig. 2). However, the lack of a significant statistical association with ARGs in rice tissues suggests that *int-I2* may not be the primary vehicle for ARG dissemination within these compartments. Additionally, *tetGF* and *tetPA*, previously reported in soil and aqueous bacterial communities (61), were detected in rice grain-associated bacteria. This finding highlights the need for further investigation into the mechanisms driving *tetGF* and *tetPA* transfer in rice systems.

The spread of *qnrB4* and *qnrD* via the phyllosphere bacteria is a major concern, as it highlights the potential risk of these genes being transferred to human pathogens, particularly through the consumption of contaminated rice. These genes are often located on plasmids, which facilitate their HGT among bacterial communities (60). The application of AG-SeNPs led to a reduction in the copy numbers of both *qnrB* and *qnrD* in the phyllosphere bacteria (Fig. 1), suggesting that Se could modulate ARG abundance, possibly through mechanisms involving the regulation of antioxidant enzyme activities (62). The interaction between T-ARGs and QA-ARGs is observed in the rice tissues, indicating that these genes not only coexist but also interact to influence resistance patterns. The expression of one gene can impact the abundance of another, either through competitive or synergistic mechanisms (26, 63). The reduction of *tetGF* facilitated the decrease in abundance of *qnrB46,47,48*, while *tetQ* and *tetR* were also reduced under these conditions. The reduction in the abundance of *qnrB46,47,48* also contributed to a decrease in *tetR*, which can decrease *qnrB4* (Fig. 3c). Thus, the abundance of the aforementioned ARGs in rice tissues is significantly reduced following AG-SeNP application.

However, the copy numbers of *qnrD* and *aac(6’)-Ib* in rice-associated microbial communities were significantly increased after the application of 15 g ha^−1^ and 60 g ha^−1^ of AG-SeNPs, implying that resistance to quinolone antibiotics continues to spread among bacterial populations. The observed U-shaped dose-response relationship between AG-SeNP application and ARG abundance suggests that low concentrations (NS1) may be insufficient to inhibit ARG-harboring microbial populations, while excessive dosing (NS3) could induce oxidative or osmotic stress, triggering microbial defense mechanisms and selectively enriching resistant taxa. There are relatively low QA-ARGs in rice under NS2 and SS2 treatment, with no significant difference in Se levels between the two rice tissues. These results underscore the necessity of optimizing AG-SeNP application rates to effectively mitigate the accumulation of ARGs in the environment.

### Mechanisms of bacteria-mediated ARG reduction under AG-SeNP application

As demonstrated through co-occurrence network analysis, the number of ARGs associated with bacterial taxa in the rhizosphere decreased from 18 (NF) to 15 (NS2), with network complexity (edges) reduced by 27.3% (Fig. 4a and b). These results indicate that the AG-SeNP application reshapes the rhizosphere microbial communities and diminishes ARG transfer among bacterial populations (64). Proteobacteria is associated with resistance to multiple types of antibiotics and known as ARG hosts (60, 65). *Ramlibacter*, commonly detected in rice straw, serves as an effective aromatic compound-degrading bacterium and effectively eliminates *sul1* and *sul2* genes (66). This study further proves that *Ramlibacter sp013087525* has the capacity to eliminate *tetM*, *tetPB*, and *tetX* (Fig. 4c). Rice growth occurs in a flooded anaerobic environment, favoring the dominance of *Mariniphaga* (a genus of anaerobic bacteria) in rhizosphere soil community. *Mariniphaga anaerophila* and *Chazhemtobacterium aquaticus* are well-known antibiotic producers and carriers of *tetGF* and *tetPA* (as observed in this study). They are widely distributed in various livestock manures, especially under aerobic conditions (65). The application of AG-SeNPs effectively reduced the abundance of these bacterial communities, resulting in a significant decline in *tetGF*. Although this process increases the potential risk of *tetPA*, its overall decreasing trend suggests that its reduction is driven by the synergistic effects of multiple microbial taxa. Moreover, *Sediminibacter magnilacihabitans*, belonging to phylum Bacteroidota, is also identified as the most frequent potential ARG hosts (67), directly contributing to the increased copy numbers of ARGs (*tetGF*, *tetX*, *tetQ*, *tetW*, *tetO*, and *tetR*) and *int-I1* in this study (Fig. 4a). *Joostella sp013391805* and *Massilia uncultured bacterium*, along with the other 18 microbial species, collectively influence the copy numbers of *tetGF*, *tetX*, *tetW*, *tetQ*, *qnrD*, and *qnrB-bob_resign*. Notably, the increased abundance of *Joostella sp013391805* promotes ARG enrichment in the soil, particularly enhancing the abundance of *tetGF*, *tetX*, *tetQ*, *tetW*, *tetO*, *tetR*, *qnrB-bob_resign*, and *int-I1*. However, *Massilia uncultured bacterium*, identified as a keystone taxon under environmental pollution or other stress conditions, strengthens the adaptation and functions of microbial communities and decreases aforementioned genes (68). ARGs are commonly associated with the phylum Firmicutes (29). The relative abundance of *Lentilactobacillus buchneri* and *Hylemonella sp001432305*, belonging to Firmicutes, is decreased, leading to a corresponding decline in the abundance of *tetX* and *tetGF* in the soil.

Zhang et al. (26) pointed out that Se could lower ARG prevalence in natural ecosystems by inhibiting bacterial selenocompound metabolism and chemotaxis pathways, which in turn alter microbial interactions and reshape ARG-host associations in the phyllosphere. The AG-SeNP application increased the complexity of associations between phyllosphere bacteria and ARGs (Fig. 4c), indicating that AG-SeNPs facilitated the establishment of additional pathways for ARG attenuation, distinct from those observed in the soil environment. There is a greater co-occurrence of bacterial communities in phyllosphere bacteria, MGEs, and ARGs in AG-SeNPs treatments compared to the NF group. The high abundance of *Hylemonella sp001432305* observed in the NF group increased the copy number of *qnrB4* (Fig. 4a). However, AG-SeNP application disrupts this pathway, leading to a reduction in *qnrB4*. The application of AG-SeNPs enhances the relative abundance of Firmicutes (*Lactobacillus hamster*, *Lactobacillus kullabergensis*, and *Alkalihalobacillus trypoxylicola*) and Actinobacteria (*Microbacterium sp900156435*, *Curtobacterium sp001742745*, and *Microbacterium enclense*), which contributes to the decline of *qnrB4* in AG-SeNP treatments compared to the NF group (Fig. 4d).

Overall, functional prediction suggests that AG-SeNP application suppresses ARG dissemination through reshaping microbial communities and modulating mechanisms such as ecological competition, metabolic inhibition, and stress-adaptive regulation. Although some antibiotic-resistant bacteria and ARGs are eliminated by AG-SeNPs, the environmental risks associated with ARGs in rice paddy ecosystems remain a concern. These ARGs can re-enter microbial cells through natural transformation under favorable conditions, conferring resistance to new hosts.

### Functional profiling of microbiota and key environmental factors shaping community structure

Functional prediction of rhizosphere bacteria reveals significant changes in three key pathways, ABC transporters, ribosome, and purine metabolism, as dominant drivers in shaping ARG dynamics (Fig. 5a). Moderate AG-SeNP application (NS2) increases ABC transporter activity, consistent with increased microbial efflux activity that can alleviate intracellular accumulation of toxicants and consequently reduce selective pressure for ARG maintenance. Additionally, the downregulation of ribosomal function and purine metabolism has suppressed protein synthesis and constrained DNA replication, thereby reducing resistance gene expression and lowering the potential for HGT among bacteria (69). Under these conditions, the total copy numbers of ARGs in NS2 decrease. Conversely, reduced ABC transporter function can weaken efflux-based resistance mechanisms, increasing bacterial susceptibility to environmental stress (NS1 and NS3 groups). Increased ribosome and purine metabolism likely facilitates the rapid proliferation of non-resistant soil bacteria, which competitively exclude ARG-carrying strains (70). This metabolic compensation may allow non-target bacteria to proliferate and potentially co-harbor ARGs, thereby undermining the resistance suppression observed under optimal SeNP dosing.

Two-component system, which is critical for environmental sensing and stress response, facilitates adaptive regulation under SeNP-induced stress in phyllosphere bacteria (Fig. 5d). By modulating antimicrobial resistance pathways, such as efflux pump regulation and quorum sensing, the enhanced two-component system can suppress ARG expression and limit HGT among phyllosphere bacteria (71). Enhanced arginine and proline metabolism reflects microbial adjustments toward greater osmotic balance and energy availability (e.g., ATP generation), which likely favors non-resistant populations and narrowed ecological niches for ARG-harboring taxa. The downregulation of photosynthesis limits primary production and carbon input, constraining microbial growth and the proliferation of potential ARG hosts (70). Consequently, the abundance of ARGs in phyllosphere bacteria is reduced.

In addition to MGEs and bacterial communities, environmental factors are critical indicators indirectly affecting ARG variations. The RDA analysis results explained 48.78% of the variations in relationships between bacterial communities and soil physicochemical properties under different regimes (Fig. 5b), indicating that SeNPs reshaped the interactions of rhizosphere microbial community and soil properties (64). Soil Se content, pH, and OM significantly influence the functional soil microbes that contribute to changes in the abundance of rhizosphere ARGs and MGEs. Among these factors, elevated soil Se levels following AG-SeNP application increase the relative abundance of *Hydrogenophaga* and *Sunxiuqinia*, which are positively associated with pH and known to degrade antibiotic-related organic acids (72, 73). Li et al. (74) demonstrated that pH exhibited a significant negative correlation with the abundance of ARGs. Soil pH modulates the selection of ARG hosts during the conjugative transfer of resistant plasmids following SeNP application (27). This process reduces the selective pressure of antibiotics on ARG hosts, decreasing copy numbers of T-ARGs. Meanwhile, decreased OM levels are associated with increased abundance of genera such as *Luteitalea* and *Ramlibacter*. The reduction in OM limits its competition with ARGs for adsorption sites, thereby inhibiting the vertical transport of certain ARGs (75).

The AG-SeNP application significantly reduces the relative abundance of *Acinetobacter* and *Frankia*, while increasing that of *Limonospira*, *Sphingomonas*, and *Microbacterium* in phyllosphere samples (Fig. 5c). *Acinetobacter*, a common bacterium widely distributed in soil environments, is known to carry multiple ARGs, contributing to ARG dissemination (76). *Frankia*, a nitrogen-fixing bacterium, has been shown to effectively reduce beta-lactam resistance genes. Therefore, reducing the relative abundance of these bacteria in the phyllosphere is an important step in ARG mitigation. Although the relationship between leaf Se and soil ARGs is non-linear, these findings collectively support a multiscale regulatory pathway in which foliar Se application alters soil environmental conditions and microbial ecology, thereby suppressing ARG propagation.

### Microbial filtering and functional enhancement drive ARG suppression

*Mariniphaga anaerophila* was newly identified as an ARG host, and its enrichment likely reflects adaptation to elevated soil EC, which imposes salt stress on microbial communities (77). Such conditions are known to favor carbon-competitive taxa that thrive under ionic stress (78), potentially outcompeting *tetPA-carrying* bacteria through resource exclusion and niche displacement. As a result, the proliferation of *Mariniphaga anaerophila* leads to an increase of *tetPA*. The increase in *Pyrinomonas methylaliphatogenes* reduces the availability of ecological niches for ARG hosts, thereby limiting the *tetGF* dissemination. The ecological role of *Pyrinomonas methylaliphatogenes* in carbon cycling is further reflected in its strong association with OM and AK (79). Under low OM conditions, its relative abundance increases, potentially occupying adsorption sites and reducing the spatial availability for ARG-carrying taxa, which may constrain vertical gene transfer (75). The observed suppression of ARG hosts may be further attributed to selenium-induced generation of reactive oxygen species, which impairs DNA and protein synthesis and selectively inhibits taxa lacking robust antioxidant defenses (80). In the phyllosphere, *P. aeruginosa* facilitates the biotransformation of selenium into less toxic forms, enabling ecological adaptation under Se exposure and contributing to its increased abundance (81). Under this condition, it alleviates Se-induced oxidative stress, as indicated by its negative association with MDA (82). *P. aeruginosa* can indirectly inhibit horizontal transfer of *tetQ* by modulating local microbial interactions, particularly with Bacteroides (83). Collectively, these microbially mediated mechanisms contributed to the observed attenuation of *tetQ* in the phyllosphere.

Analysis of microbial assembly processes revealed that rhizosphere communities were predominantly shaped by stochastic mechanisms across all treatments, likely due to nutrient enrichment from fertilization, which buffered environmental selection and weakened the role of deterministic selection (44). In contrast, the phyllosphere exhibited stronger deterministic assembly, reflecting selective constraints imposed by the plant surface microenvironment. Notably, AG-SeNP application intensified both homogeneous and heterogeneous selection, suggesting enhanced environmental filtering and niche differentiation. This deterministic shift likely contributed to the exclusion of ARG-carrying taxa and simplification of microbial interactions, thereby reinforcing ARG attenuation through community-level restructuring. These findings suggest that phyllosphere community assembly represents a potentially important control point for modulating the persistence of ARG-associated taxa under foliar interventions.

The SEM model revealed that EC plays an important downstream role, mediating the effects of foliar Se application on soil ARG dynamics (Fig. 6d). Elevated EC imposes osmotic stress on microbial cells, suppresses proton-motive force-driven resistance systems, and reshapes microbial community composition away from ARG hosts (84).

Alkyl glycoside-stabilized selenium nanoparticles enhance Se biofortification in crops and mitigate the dissemination of ARGs by reshaping microbial communities and regulating soil properties. This dual functionality aligns with the goals of green nanotechnology in agriculture, which aims to support sustainable crop production and food safety by reducing the environmental burden of antibiotic resistance.

### Conclusions and future perspectives

Alkyl glycoside-stabilized selenium nanoparticles applied at 30 g ha⁻^1^ can both enhance Se biofortification in rice and significantly reduce the abundance of antibiotic resistance genes across the rhizosphere, phyllosphere, and grains. *TetPA* and *tetGF* were associated with shifts in microbial community structure and key metabolic pathways, which alleviated selection pressure and limited HGT. Soil pH and OM regulated ARG abundance by restructuring microbial communities, whereas Se enrichment induced deterministic assembly within the phyllosphere and suppressed ARGs via modulation of MGEs, with EC acting as a key mediator. By providing a viable strategy to reduce the agricultural reservoir of ARGs, this work contributes to the One Health objective of curbing resistance transmission across environmental and food-chain interfaces.

Translating AG-SeNPs into a practical agricultural intervention requires further validation through field trials across diverse paddy systems and long-term ecological safety assessments. Future efforts should focus on optimizing dosage under varying edaphic and climatic conditions, integrating AG-SeNPs with complementary soil or nutrient management practices, and extending this strategy to other crops with similar root-soil-phyllosphere interfaces. Building on the principles of sustainable nano-carrier design, future research should also explore encapsulating Se within bio-based fibrous matrices. Such engineered architectures could significantly enhance the spatiotemporal control of Se release in paddy soils, thereby improving the precision and efficacy of ARG mitigation.

## Data Availability

All 16S rRNA gene sequencing reads have been deposited in the NCBI Sequence Read Archive (SRA) under BioProject accession number PRJNA1392298. Other data supporting the findings of this study are available within the article and its supplemental material.

## SUPPLEMENTAL MATERIAL

Supplemental material aem.02231-25-s0001.docx Tables S1 and S2; Fig. S1 to S4.

ASM does not own the copyrights to Supplemental Material that may be linked to, or accessed through, an article. The authors have granted ASM a non-exclusive, world-wide license to publish the Supplemental Material files. Please contact the corresponding author directly for reuse.
