# Supplementary material for: Nano-selenium mitigates antibiotic resistance in paddy ecosystems via microbiome remodeling and environmental filtering shifts
Source: Appl Environ Microbiol. 2026 Feb 27;92(3):e02231-25. doi: 10.1128/aem.02231-25 (PMC12997761; doi:10.1128/aem.02231-25)
Supplement: Supplemental material — Tables S1 and S2; Fig. S1 to S4. [file aem.02231-25-s0001.docx]

Nano-selenium mitigates antibiotic resistance in paddy ecosystems via microbiome remodeling and environmental filtering shifts

Xiaorong Zhang^a,b^, Qiaobing Luo^a,b,c^, Zongqiang Gong^a,b*^, Huimin Yang^a,b,d^, Xin Chen^e^, Boshi Wang^a,b,f^, Meng Yuan^a,b,f^, Yue Chen^a,b,d^, Yanjie Jia^a,b,d^, Shuhai Guo^b^

Corresponding author: Zongqiang Gong (zgong@iae.ac.cn)

Table S1 Primer pairs used in Smartchip for specific detection and quantification of ARGs.

| Target gene | Primer | Sequence |
| --- | --- | --- |
| *tet32* | *tet32*-F | CCATTACTTCGGACAACGGTAGA |
|  | *tet32*-R | CAATCTCTGTGAGGGCATTTAACA |
| *tet36* | *tet36-F* | AGAATACTCAGCAGAGGTCAGTTCCT |
|  | *tet36-R* | TGGTAGGTCGATAACCCGAAAAT |
| *tet44* | *tet44*-F | CTCATGTAGATGCAGGAAAGACG |
|  | *tet44*-R | GTAACTGCTGCCTGAATTGTGA |
| *tetB* | *tet*M-F | AGTGCGCTTTGGATGCTGTA |
|  | *tet*M-R | AGCCCCAGTAGCTCCTGTGA |
| *tetC* | *tetC*-F | ACTGGTAAGGTAAACGCCATTGTC |
|  | *tetC*-R | ATGCATAAACCAGCCATTGAGTAAG |
| *tetE* | *tetE*-F | TTGGCGCTGTATGCAATGAT |
|  | *tetE*-R | CGACGACCTATGCGATCTGA |
| *tetH* | *tetH*-F | TTTGGGTCATCTTACCAGCATTAA |
|  | *tetH*-R | TTGCGCATTATCATCGACAGA |
| *tetJ* | *tetJ*-F | CAGCGCCCATACGCCATTTA |
|  | *tetJ*-R | CCTACTTCAGTAGTGTGCCAAGC |
| *tetM* | *tetM*-F | GGAGCGATTACAGAATTAGGAAGC |
|  | *tetM*-F | TCCATATGTCCTGGCGTGTC |
| *tetO* | *tet*O-F | CAACATTAACGGAAAGTTTATTGTATACCA |
|  | *tet*O-R | TTGACGCTCCAAATTCATTGTATC |
| *tetQ* | *tet*Q-F | CGCCTCAGAAGTAAGTTCATACACTAAG |
|  | *tet*Q-R | TCGTTCATGCGGATATTATCAGAAT |
| *tetR* | *tetR*-F | CCGTCAATGCGCTGATGAC |
|  | *tetR*-R | GCCAATCCATCGACAATCACC |
| *tetS* | *tetS*-F | TTAAGGACAAACTTTCTGACGACATC |
|  | *tetS*-R | TGTCTCCCATTGTTCTGGTTCA |
| *tetT* | *tetT*-F | CCATATAGAGGTTCCACCAAATCC |
|  | *tetT*-R | TGACCCTATTGGTAGTGGTTCTATTG |
| *tetW* | *tetW-*F | ATGAACATTCCCACCGTTATCTTT |
|  | *tetW-*Q | ATATCGGCGGAGAGCTTATCC |
| *tetX* | *tet*X-F | AAATTTGTTACCGACACGGAAGTT |
|  | *tet*X-R | CATAGCTGAAAAAATCCAGGACAGTT |
| *tetbP* | *tetbP*-F | TGGGCGACAGTAGGCTTAGAA |
|  | *tetbP*-R | TGACCCTACTGAAACATTAGAAATATACCT |
| *tetGF* | *tetG*-F | TCGCGTTCCTGCTTGCC |
|  | *tetG*-R | CCGCGAGCGACAAACCA |
| *tetPA* | *tetPA*-F | GGAGCGATTACAGAATTAGGAAGC |
|  | *tetPA*-R | TCCATATGTCCTGGCGTGTC |
| *tetPB* | *tet*PB-F | TGGCAAGACGAGTTTGACTGA |
|  | *tet*PB-R | GATCGCTCCACTTCAGCGATAA |
| *aac(6')-Ib* | *aac(6')-Ib*-F | CGTCGCCGAGCAACTTG |
|  | *aac(6')-Ib*-R | CGGTACCTTGCCTCTCAAACC |
| *oqxA* | *oqxA*-F | GAGTCAACCTACCTCCACTATCA |
|  | *oqxA*-R | GCTGCGAGTTATCCAGCAG |
| *qnrA* | *qnrA*-F | AGGATTTCTCACGCCAGGATT |
|  | *qnrA*-R | CCGCTTTCAATGAAACTGCAA |
| *qnrB4* | *qnrB4*-F | TCACCACCCGCACCTG |
|  | *qnrB4*-R | GGATATCTAAATCGCCCAGTTCC |
| *qnrB-bob_resign* | *qnrB-bob_resign*-F | GCGACGTTCAGTGGTTCAGA |
|  | *qnrB-bob_resign*-R | GCTGCTCGCCAGTCGAA |
| *qnrB46,47,48* | *qnrB46,47,48*-F | CGACGTTCAGTGGTTCAGATCTC |
|  | *qnrB46,47,48*-R | GCCAAGCCGCTCCATGAG |
| *qnrD* | *qnr*D-F | CGCTGGAATGGCACTGTGA |
|  | *qnr*D-R | GCTCTCCATCCAACTTCACTCC |
| *qnrS1_S3_S5* | *qnrS1_S3_S5*-F | CCACTTTGATGTCGCAGATCTTC |
|  | *qnrS1_S3_S5*-R | CCCTCTCCATATTGGCATAGGAAA |
| *qnrS2* | *qnrS2*-F | TCCCGAGCAAACTTTGCCAA |
|  | *qnrS2*-R | GGTGAGTCCCTATCCAGCGA |
| *QnrVC1_VC3_VC6* | *QnrVC1_VC3_VC6*-F | CTCACATCAGGACTTGCAAGAA |
|  | *QnrVC1_VC3_VC6*-R | ATGAAGCATCTCGAAGATCAGC |
| *QnrVC4_VC5_VC7* | *QnrVC4_VC5_VC7*-F | TTCCTTTAAACGGGCAAACCTC  CGATACCTGATTCATGAAGCTAGC |
|  | *QnrVC4_VC5_VC7*-R |  |
| *int-I1* | *int-I1*-F | GGCATCCAAGCAGCAAG |
|  | *int-I1*-R | AAGCAGACTTGACCTGA |
| *int-I2* | *int-I2*-F | TGCTTTTCCCACCCTTACC |
|  | *int-I2*-R | GACGGCTACCCTCTGTTATCTC |

Table S2. Sequencing statistics of full-length 16S rRNA gene amplicon sequencing for all samples.

| Sampling type | Treatment group | Sequences | Bases (bp) | Average length(bp) |
| --- | --- | --- | --- | --- |
| Soil sample | NF1 | 31127 | 45285390 | 1454.86 |
|  | NF2 | 35241 | 51276781 | 1455.03 |
|  | NF3 | 38253 | 55517690 | 1451.33 |
|  | BF1 | 41094 | 59701636 | 1452.81 |
|  | BF2 | 39312 | 57121032 | 1453.02 |
|  | BF3 | 39204 | 56983295 | 1453.51 |
|  | SS1 | 37714 | 54807362 | 1453.24 |
|  | SS2 | 37883 | 55053368 | 1453.25 |
|  | SS3 | 33871 | 49205101 | 1452.72 |
|  | NS1-1 | 35317 | 51216227 | 1450.19 |
|  | NS1-2 | 38803 | 56327401 | 1451.62 |
|  | NS1-3 | 37679 | 54595242 | 1448.96 |
|  | NS2-1 | 31218 | 45408874 | 1454.57 |
|  | NS2-2 | 39249 | 57055599 | 1453.68 |
|  | NS2-3 | 37481 | 54473345 | 1453.36 |
|  | NS3-1 | 37582 | 54694793 | 1455.35 |
|  | NS3-2 | 39328 | 57159603 | 1453.41 |
|  | NS3-3 | 37487 | 54541755 | 1454.95 |
| Phyllosphere sample | NF1 | 31038 | 45645187 | 1470.62 |
|  | NF2 | 34095 | 50214531 | 1472.78 |
|  | NF3 | 31619 | 46964648 | 1485.33 |
|  | BF1 | 33912 | 49920833 | 1472.07 |
|  | BF2 | 32420 | 49029350 | 1512.32 |
|  | BF3 | 34351 | 52412346 | 1525.79 |
|  | SS1 | 32689 | 47069509 | 1439.92 |
|  | SS2 | 34265 | 49039504 | 1431.18 |
|  | SS3 | 39757 | 57134687 | 1437.1 |
|  | NS1-1 | 39516 | 58875002 | 1489.9 |
|  | NS1-2 | 33192 | 49194013 | 1482.1 |
|  | NS1-3 | 30700 | 46926303 | 1528.54 |
|  | NS2-1 | 35492 | 50737505 | 1429.55 |
|  | NS2-2 | 33295 | 48750445 | 1464.2 |
|  | NS2-3 | 32801 | 47128488 | 1436.8 |
|  | NS3-1 | 30343 | 44286099 | 1459.52 |
|  | NS3-2 | 32025 | 46992834 | 1467.38 |
|  | NS3-3 | 30891 | 45880717 | 1485.25 |


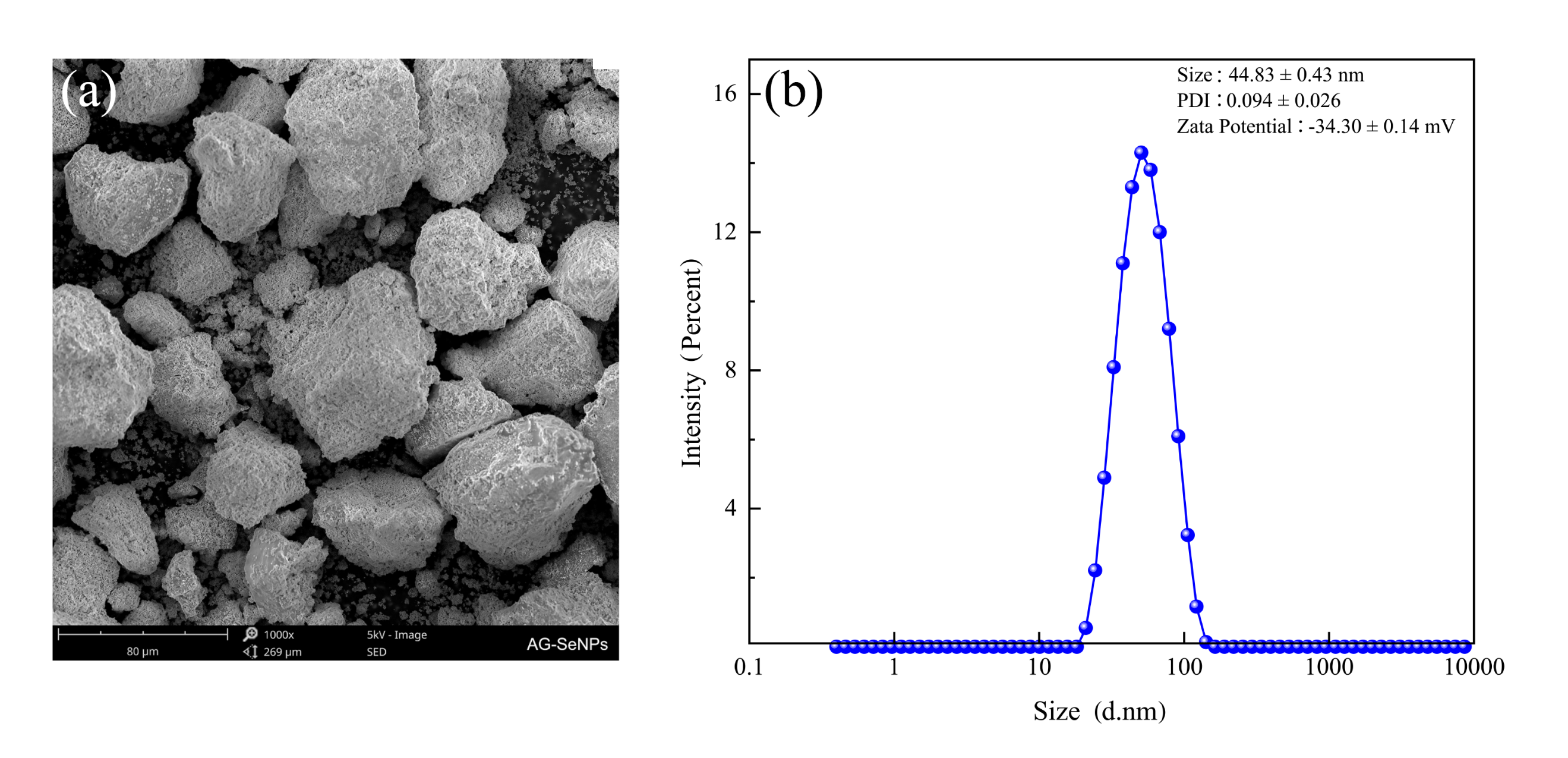


Fig. S1 Scanning electron microscopy (a) and dynamic light scattering-based particle size distribution (b) of AG-SeNPs.


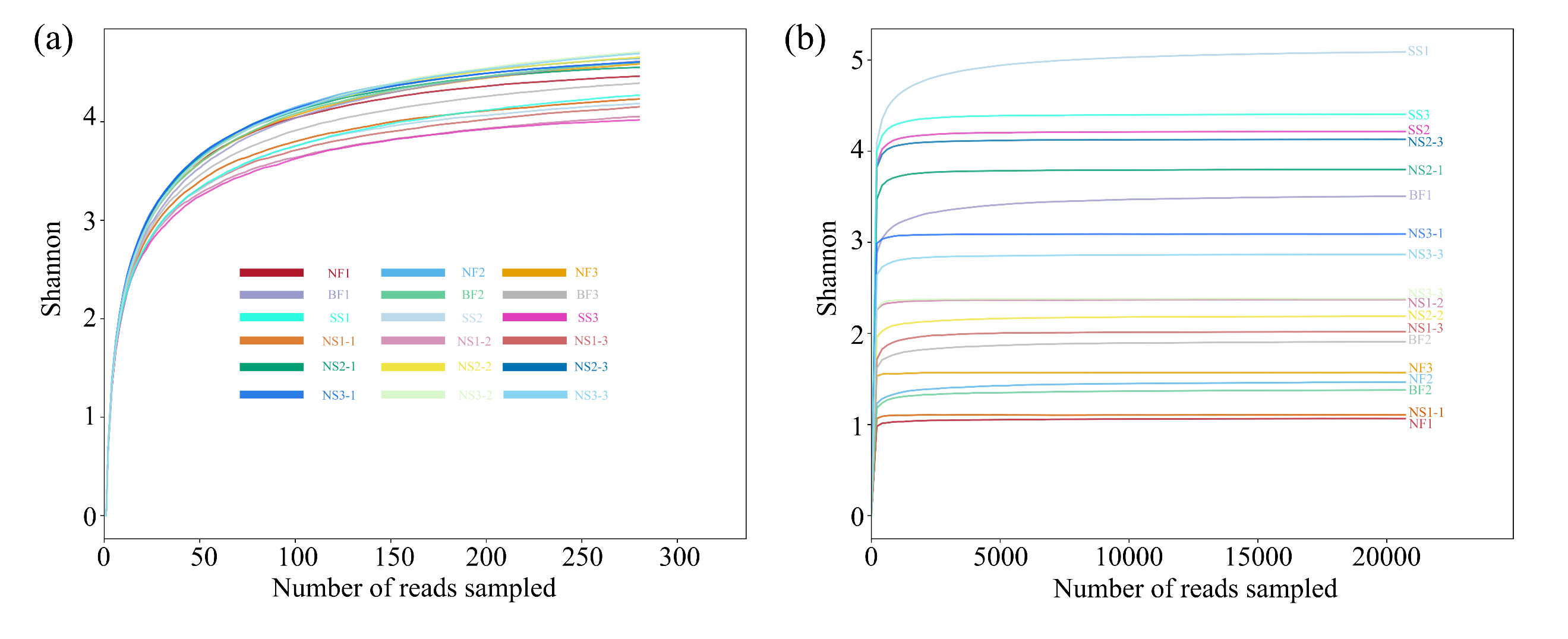


Fig. S2 Shannon diversity–based rarefaction curves of bacterial communities in the (a) rhizosphere soil and (b) phyllosphere samples based on full-length 16S rRNA gene sequencing.


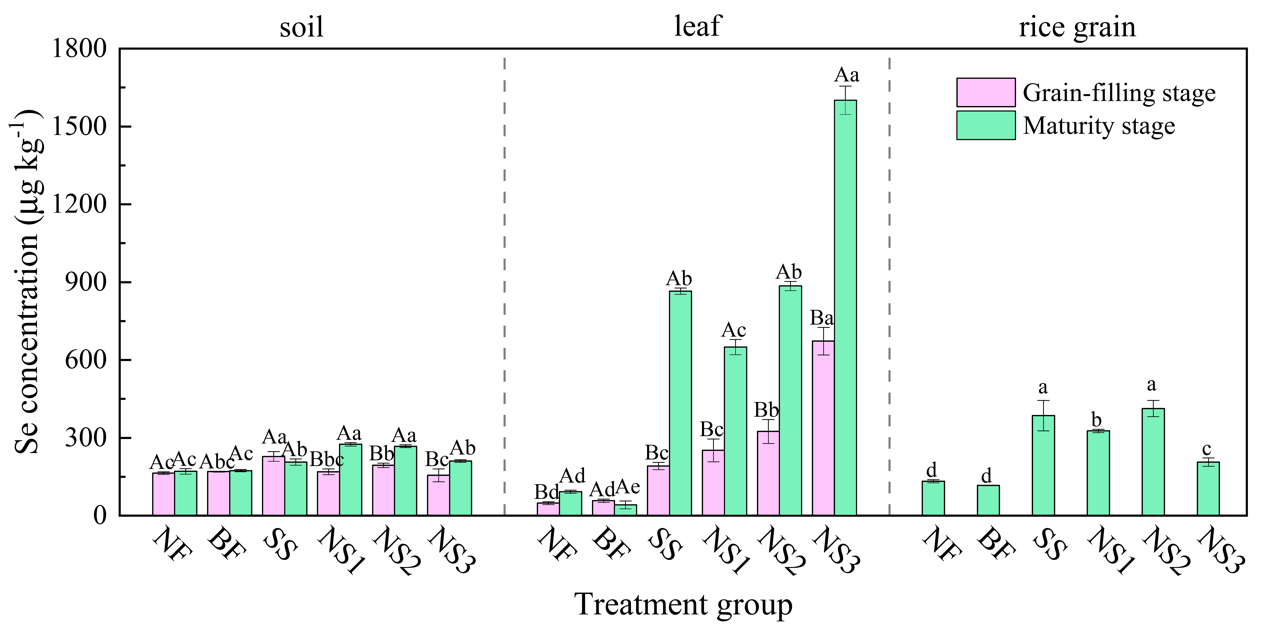


Fig. S3 Se concentration in soil, leaves, and rice grains.

Note: Different lowercase letters indicate significant differences among treatments, and different uppercase letters indicate significant differences within the same treatment (P < 0.05).


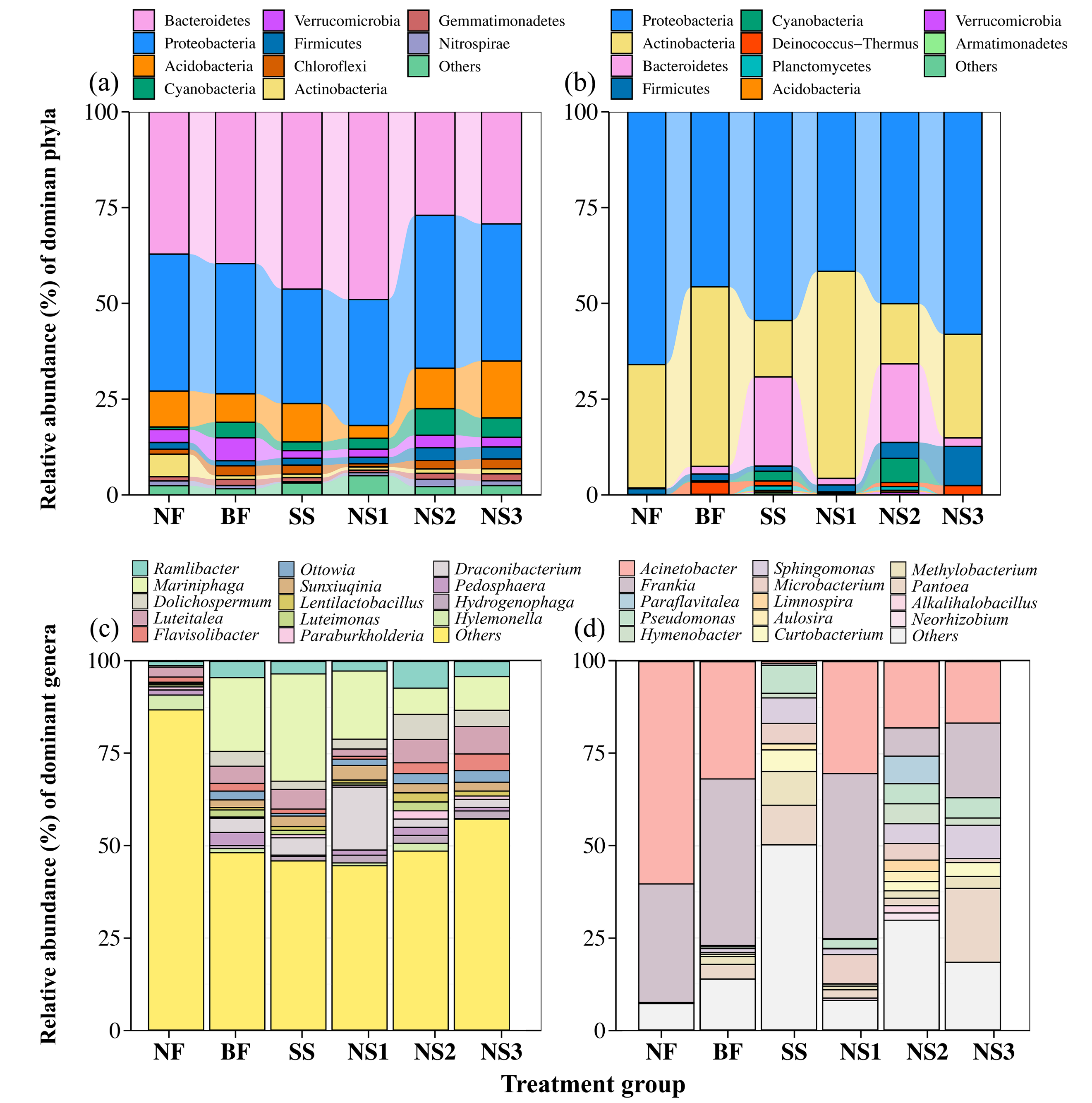


Fig. S4 Relative abundance of bacterial taxa at the (a) phylum level in soil, (b) phylum level in the phyllosphere, (c) genus level in soil, and (d) genus level in the phyllosphere.
